# Ischemic Stroke Risk Factors in Patients with Atrial Fibrillation Treated with New Oral Anticoagulants

**DOI:** 10.3390/jcm10061223

**Published:** 2021-03-16

**Authors:** Paweł Wańkowicz, Jacek Staszewski, Aleksander Dębiec, Marta Nowakowska-Kotas, Aleksandra Szylińska, Iwona Rotter

**Affiliations:** 1 Department of Medical Rehabilitation and Clinical Physiotherapy, Pomeranian Medical University in Szczecin, Żołnierska 48, 71-210 Szczecin, Poland; aleksandra.szylinska@gmail.com (A.S.); iwrot@wp.pl (I.R.); 2 Department of Neurology, Military Medical Institute, Warszawa, Szaserów 128, 04-141 Warszawa, Poland; jacekstaszewski@wp.pl (J.S.); adebiec@wim.mil.pl (A.D.); 3 Department of Neurology, Medical University of Wrocław, Borowska 213, 50-566 Wrocław, Poland; marnow64@interia.pl

**Keywords:** ischemic stroke, risk factor, atrial fibrillation, new oral anticoagulants

## Abstract

The most commonly used therapeutic option for the prevention of ischemic stroke in patients with atrial fibrillation is new- or old-generation oral anticoagulants. New oral anticoagulants are at least as effective as old-generation oral anticoagulants in the prevention of ischemic stroke, with a reduced risk of life-threatening hemorrhage. Moreover, the constant monitoring of these drugs in the patient’s blood is not required during routine use. However, ischemic stroke can still occur in these patients. Therefore, the aim of this study was to investigate the pattern of risk factors for ischemic stroke in patients with atrial fibrillation treated with new oral anticoagulants. Our multicenter retrospective study involved 2032 patients with acute ischemic stroke. The experimental group consisted of 256 patients with acute ischemic stroke and nonvalvular atrial fibrillation, who were treated with new oral anticoagulants. The control group consisted of 1776 ischemic stroke patients without coexisting atrial fibrillation. The results of our study show that patients with atrial fibrillation treated with new oral anticoagulants are more likely to display thrombotic, proatherogenic, and proinflammatory factors in addition to the embolic factors associated with atrial fibrillation. Therefore, solely taking new oral anticoagulants is insufficient in protecting this group of patients from ischemic stroke.

## 1. Introduction

Atrial fibrillation (AF) is the most common cardiac arrhythmia found in everyday clinical practice. Currently, the prevalence of AF in the global population ranges from 4 to 15 percent depending on the age of the patient [1,2,3]. This number is expected to double in the coming years due to the increasing life span of the general population, as well as the development of new techniques allowing faster detection of previously undiagnosed cases of AF [4,5].

The late detection or inadequate treatment of AF may result in serious complications, such as ischemic stroke. AF may account for up to 30% of cerebral ischemic strokes [6,7,8]. The most commonly used therapeutic option for the prevention of embolic complications in patients with AF is oral anticoagulants.

New oral anticoagulants (NOACs) are at least as effective as vitamin K antagonists (VKA) in the primary and secondary prevention of ischemic stroke, with a reduced risk of life-threatening hemorrhages—especially intracranial—as is confirmed in numerous studies [9,10,11,12]. Moreover, the constant monitoring of these drugs in the patient’s blood is not required during routine use. However, data suggest that although the use of NOACs may be effective in preventing ischemic stroke associated with AF, a considerable number of patients still suffer from strokes [13,14].

Over the years, a number of factors have been identified as predisposing to the development of AF. Some of these are non-modifiable factors such as age, gender, genetic predisposition, or ethnicity. Others are modifiable—hypertension, coronary artery disease, heart failure, diabetes, or atherosclerosis [15,16,17,18,19]. Importantly, these AF risk factors are also risk factors for ischemic stroke [20]. Therefore, the aim of this study was to investigate the pattern of non-modifiable and modifiable risk factors for ischemic stroke in patients with nonvalvular atrial fibrillation (NVAF) treated with NOACs.

## 2. Materials and Methods

This was a multicenter retrospective study conducted between 2014 and 2020 at the Department of Neurology of the Pomeranian Medical University in Szczecin, the Department of Neurology of the Military Medical Institute in Warsaw, and the Department of Neurology of the Medical University of Wrocław in Poland.

The study included 2032 consecutive patients with acute ischemic stroke. Patients were divided into two groups. The experimental group consisted of 256 patients with acute ischemic stroke and nonvalvular atrial fibrillation. These patients had been treated with NOACs for the prevention of ischemic stroke. Because of the size of the study group, specific types of NOAC were not distinguished. The control group consisted of 1776 ischemic stroke patients without coexisting atrial fibrillation.

In accordance with international guidelines, all patients with ischemic stroke underwent neurological examination, electrocardiogram (ECG), diagnostic neuroimaging (computed tomography or magnetic resonance imaging of the brain), and vascular examination (Doppler ultrasound/angio-computed tomography/angio-magnetic resonance imaging of the carotid and vertebral arteries) [21]. All types of ischemic stroke were included in the study.

Data on the most common risk factors for ischemic stroke were collected by interview and through the analysis of medical records. These risk factors included baseline demographics such as sex, age, and comorbidities—for example, AF treated with NOACs, hypertension, diabetes mellitus, dyslipidemia, coronary artery disease, hemodynamically significant internal carotid artery (ICA) stenosis or ICA occlusion, peripheral arterial disease, previous stroke, and cigarette smoking. The Bioethics Committee of the Pomeranian Medical University issued consent to conduct the study (KB-0012/49/07/2020/Z).

The diagnosis of AF was based on the absence of P waves with the isoelectric line replaced by irregular high-frequency oscillations (f waves) in an ECG, either taken on admission to the neurology department or from the patient’s medical records, which included information on the intake of NOACs. We also took into account patients who reported the presence of AF during the interview [22]. The diagnosis of hypertension was based on medical history and intake of hypotensive medications. Hypertension was defined by systolic blood pressure ≥140 mm Hg and/or diastolic blood pressure ≥90 mm Hg in repeated tests [23].

The diagnosis of diabetes mellitus was based on the patient’s history, analysis of medical records, and intake of glucose-lowering medications. Diabetes mellitus was defined as a level of fasting blood glucose ≥126 mg/dL after a minimum of two tests, or a glucose level of ≥200 mg/dL measured at any time during the day [24].

The diagnosis of dyslipidemia was based on the interview, analysis of patient medical records, and intake of lipid-lowering medications. Dyslipidemia was defined as a serum cholesterol concentration of >190 mg/dL, low-density lipoprotein cholesterol >115 mg/dL, serum triglyceride concentration >150 mg/dL, and high-density lipoprotein cholesterol <40 mg/dL in males and <45 mg/dL in females [25].

The diagnosis of coronary heart disease was based on the interview, analysis of the patient’s medical records, or intake of cardiovascular medications.

The diagnosis of ICA hemodynamically significant stenosis was based on medical records and/or vascular examination (Doppler ultrasound/angio-computed tomography/angio-magnetic resonance imaging) where the degree of stenosis was 50% or greater [26,27].

The diagnosis of peripheral arterial disease was based on the interview and an analysis of medical records.

The determination of cigarette smoking was based on the interview and medical records. Smoking was defined as current smoking of any number of cigarettes.

The diagnosis of recurrent stroke was based on patient history according to medical records or if the patient, caregiver, or relative reported this diagnosis.

### Statistical Analysis

The null hypothesis was that there would be no differences in risk factors between ischemic stroke patients with NVAF who took NOACs and ischemic stroke patients without AF. The alternative hypothesis was that there would be significant differences in risk factors between ischemic stroke patients with NVAF who took NOACs and ischemic stroke patients without AF. In order to compare the characteristics of both groups, the Mann–Whitney U test was used for quantitative data. Fisher’s exact test was used for qualitative data. Multiple logistic regression was calculated in order to assess the odds ratio of independent risk factors for both groups. Differences were considered significant at *p* < 0.05. All computations were performed using licensed Statistica 13.0 software (StatSoft, Tulsa, OK, USA).

## 3. Results

In total, 256 (12.6%) patients of the 2032 acute ischemic stroke cohort included in this study had a diagnosis of NVAF and were taking NOACs. The median age of all patients was 71 years (69.9 ± 8.7 years). In total, 958 patients (47.15%) were male. Hypertension was present in 1543 patients (75.94%), diabetes mellitus in 685 (33.71%), dyslipidemia in 876 (43.11%), ICA significant stenosis/occlusion in 298 (14.67%), coronary heart disease in 873 (42.96%), peripheral arterial disease in 262 (12.89%), previous stroke in 954 (46.95%), and cigarette smoking in 1041 (51.23%).

### 3.1. Comparison of Risk Factors for Ischemic Stroke in Patients with Nonvalvular Atrial Fibrillation Treated with New-Generation Oral Anticoagulants versus Patients without Atrial Fibrillation

A comparison of both groups is shown in Table 1.

### 3.2. Analysis of Risk Factors for Ischemic Stroke in Patients with Nonvalvular Atrial Fibrillation Treated with a New Oral Anticoagulant and Patients without Atrial Fibrillation

A univariate and multivariate logistic regression model is shown in Table 2.

## 4. Discussion

The most common therapeutic option for the prevention of ischemic stroke in patients with AF is treatment with VKA or NOACs. Chronic use of oral anticoagulants reduces the risk of ischemic stroke by 64% [28]. Nevertheless, this does not completely prevent the risk of ischemic stroke in these patients. In VKA treatment, one popular explanation for this is that the patient did not follow the physician’s recommendations regarding the frequency of administration, resulting in nontherapeutic international normalized ratio (INR). However, this cannot be the case for NOACs, which do not require constant monitoring of blood levels.

The results of some reports suggest that the sole use of anticoagulants for AF patients is insufficient in significantly reducing the risk of stroke, due to the presence of other factors that often accompany AF. In a study by Tokunaga et al. on AF patients, prior use of warfarin, an old-generation oral anticoagulant, was associated with a higher risk of ischemic events in the two years following therapy when compared with no such treatment [29]. Evans et al. observed that, among AF patients who had an ischemic stroke, there was a higher rate of recurrent lacunar stroke than cardioembolic stroke, which suggests that, although the incidence of one type of stroke associated with AF may be reduced, the presence of other factors in the patient may result in a different type of stroke [30]. Many studies have also considered genetic variability as an expression of increased susceptibility to stroke in AF patients taking old-generation anticoagulants. It is apparent that the majority of patients, despite switching to a NOAC, developed ischemic stroke nonetheless [31,32,33,34].

The results of our study showed that, in addition to the embolic factor linked directly to AF, patients with this condition who were taking NOACs and suffered ischemic stroke had higher odds of developing hypertension, diabetes mellitus, dyslipidemia, and cigarette smoking compared to patients without AF—factors that favor thrombogenic, proatherogenic, and proinflammatory mechanisms.

Although it is well known that hypertension is a major risk factor for ischemic stroke, the complexity of cerebrovascular problems associated with hypertension is generally underestimated. Hypertension causes structural vascular changes that increase peripheral resistance, even with a relaxed vascular bed, which predisposes patients to distal ischemia resulting from stenosis or occlusion.

Hypertension also causes degenerative changes in the small intracerebral arteries, which can lead to extravasation and the development of focal cerebral edema, as well as lacunar infarcts. The last classic atherosclerotic mechanism leads to transient cerebral ischemic attacks or cerebral ischemic stroke via embolism or hemodynamic perfusion abnormalities [35].

Diabetes mellitus is a model of chronic vascular disease. It induces changes in microcirculation through the synthesis of extracellular matrix proteins and a thickening of the capillary basement membrane. These changes, in combination with advanced glycation end-products, oxidative stress, and inflammation, can lead to macroangiopathy. The risk for ischemic stroke in patients with diabetes is closely correlated with the severity and type of diabetes [36].

Cigarette smoking is an extremely important and independent risk factor for ischemic stroke, as confirmed by numerous studies. Probable mechanisms increasing the risk of ischemic stroke in smokers are numerous and include increased platelet aggregation, increased fibrinogen levels, and the direct effects of 1,3-Butadiene in tobacco smoke. These mechanisms accelerate atherosclerotic processes. Interestingly, it has been suggested that the risk of ischemic stroke in the smoking population is related to a chronic inflammation that leads to advanced atherosclerosis of the intracerebral and cerebral vessels [37,38].

The role of dyslipidemia in the pathogenesis of ischemic stroke, although initially unclear, is now given more credence. Dyslipidemia is a modifiable risk factor for vasculopathy [39].

Our study also showed that patients taking NOACs were significantly more likely to have non-modifiable factors—namely older age and female gender—than patients without AF.

Aging is associated with a multitude of changes in the hemostatic system. Increased procoagulant activity and a prothrombotic state are inherent in aging, through increased concentrations of procoagulant factors, thrombin formation, and platelet activation. Aging is also associated with increased levels of fibrin and fibrinogen degradation products [40,41]. All of these changes can be considered aspects of physiological aging or are provoked by other abnormalities such as chronic inflammation, malnutrition, and decreased physical activity.

Our study had several limitations. First, it was a retrospective study. Second, reference to specific types of NOAC was not possible because of the limited number of cases. Third, the time of the last administration of a NOAC was unknown in many cases. Fourth, we were unable to determine whether patients previously treated with a NOAC had their dose adjusted according to their age, weight, and creatinine levels. Fifth, only the most common risk factors for ischemic stroke were considered. Sixth, we had no information about therapy, especially those that might affect AF, such as angiotensin-converting-enzyme inhibitors, beta-blockers, and statins. Seventh, we had no information about data regarding some critical risk factors of ischemic stroke: diet (high in sodium, high in red meat, low in fruits, low in vegetables, low in fiber, and low in whole grains), alcohol consumption (any dosage), low physical activity, high BMI, high fasting plasma glucose, high systolic blood pressure, high low-density lipoprotein cholesterol, and kidney dysfunction, as measured by low glomerular filtration rate (GFR).

However, our study had some strengths. We analyzed a large group of patients with ischemic stroke that occurred during NOAC treatment. To the best of our knowledge, the risk factor profile of patients with ischemic stroke with NVAF who take NOAC has not been previously studied or reported on. Our study showed that in addition to the embolic factor linked directly to AF, patients with this condition who were taking NOACs were significantly more likely to have factors that favor thrombogenic, proatherogenic, and proinflammatory mechanisms than patients without AF. NOAC treatment is insufficient in protecting this group of patients against ischemic stroke. We presume that combination strategies, adding antiplatelet therapy or left atrial appendage occlusion to oral anticoagulation, might be helpful for this group of patients, but this requires further investigation in controlled trials.

## 5. Conclusions

The results of our study show that patients with NVAF treated with NOACs are more likely to display thrombotic, proatherogenic, and proinflammatory factors in addition to the embolic factors associated with AF. Therefore, solely ingesting NOAC is insufficient in protecting this group of patients from ischemic stroke. In light of the aforementioned data, primary or secondary prevention of ischemic stroke in this group of patients requires a modification of lifestyle, better health education, increased attention to therapeutic recommendations, and regular exercise, even at minimal intensity.

## Figures and Tables

**Table 1:** Baseline characteristics of patients with ischemic stroke with nonvalvular atrial fibrillation treated with new oral anticoagulants and patients without atrial fibrillation.

Parameter	AF NOAC (*n* = 256)	No-AF (*n* = 1776)	*p*-Value
Age, mean ± SD; Me	78.23 ± 7.32; 79.0	68.66 ± 8.41; 69.0	<0.001
Female, *n* (%)	181 (70)	893 (50)	<0.001
Comorbidities, *n* (%)			
Diabetes mellitus	134 (52)	551 (31)	<0.001
Dyslipidemia	152 (59)	724 (40)	<0.001
Hypertension	237 (92)	1306 (73)	<0.001
ICA significant stenosis/occlusion	43 (16)	255 (14)	0.302
Coronary heart disease	166 (64)	707 (39)	<0.001
Peripheral arterial disease	51 (19)	211 (11)	<0.001
Smoking	151 (58)	890 (50)	0.008
Previous stroke	161 (62)	793 (44)	<0.001

Legend: *n*—number of patients, SD—standard deviation, Me—median, AF—atrial fibrillation, ICA —internal carotid artery, NOAC—nonvalvular atrial fibrillation treated with new oral anticoagulants.

**Table 2:** Analysis of risk factors of ischemic stroke in patients with atrial fibrillation, who suffered a stroke despite NOAC therapy, compared to patients without AF (multivariate logistic regression).

Factors		Univariable Logistic Regression Models				Multivariable Logistic Regression Model *			
		OR	Cl 95%	Cl 95%	*p*	OR	Cl 95%	Cl 95%	*p*
Age		1.16	1.14	1.18	<0001	1.22	1.18	1.25	<0.001
Gender	Female	2.38	1.79	3.17	<0.001	1.93	1.37	2.70	<0.001
	Male	1.00				1.00			
Diabetes mellitus	Yes	2.44	1.87	3.18	<0.001	1.90	1.38	2.63	<0.001
	No	1.00				1.00			
Dyslipidemia	Yes	2.12	162	2.77	<0.001	2.48	1.80	3.42	<0.001
	No	1.00				1.00			
Hypertension	Yes	4.48	2.78	7.24	<0.001	4.20	2.48	7.10	<0.001
	No	1.000				1.00			
Coronary heart disease	Yes	2.78	2.12	3.66	<0.001	1.79	1.27	2.51	0.001
	No	1.00				1.00			
Peripheral arterial disease	Yes	1.84	1.31	2.58	<0.001	1.11	0.72	1.71	0.634
	No	1.00				1.00			
Smoking	Yes	1.43	1.09	1.86	0.008	2.67	1.91	3.74	<0.001
	No	1.00				1.00			
Previous stroke	Yes	2.10	1.60	2.75	<0.001	0.39	0.26	0.60	<0.001
	No	100				1.00			

Legend: OR—odds ratio, CI—confidence interval. * Note: Model was adjusted for the following variables: age, sex, diabetes mellitus, dyslipidemia, hypertension, coronary heart disease, peripheral arterial disease, smoking, previous stroke.

## Data Availability

All data that support the findings of this study are available upon request from the corresponding author.
